# Optical EUS Activation to Relax Sensitized Micturition Response

**DOI:** 10.3390/life13101961

**Published:** 2023-09-25

**Authors:** Jin-Ki Hong, Hyuk-June Moon, Hyun-Joon Shin

**Affiliations:** Bionics Research Center, Korea Institute of Science and Technology, Seoul 02792, Republic of Korea; hongjk@kist.re.kr (J.-K.H.); crescent@kist.re.kr (H.-J.M.)

**Keywords:** external urethral sphincter, striated muscle, optogenetics, micturition, continence

## Abstract

This study aims to activate the external urethral sphincter (EUS), which plays a critical role in micturition control, through optogenetics and to determine its potential contribution to the stabilization of sensitized micturition activity. The viral vector (*AAV2/8-CMV-hChR2(H134R)-EGFP*) is utilized to introduce light-gated ion channels (hChR2/H134R) into the EUS of wild-type *C57BL/6* mice. Following the induction of sensitized micturition activity using weak acetic acid (0.1%) in anesthetized mice, optical stimulation of the EUS muscle tissue expressing channel rhodopsin is performed using a 473 nm laser light delivered through optical fibers, and the resulting changes in muscle activation and micturition activity are examined. Through EMG (electromyography) measurements, it is confirmed that optical stimulation electrically activates the EUS muscle in mice. Analysis of micturition activity using cystometry reveals a 70.58% decrease in the micturition period and a 70.27% decrease in the voiding volume due to sensitized voiding. However, with optical stimulation, the micturition period recovers to 101.49%, and the voiding volume recovered to 100.22%. Stimulation of the EUS using optogenetics can alleviate sensitized micturition activity and holds potential for application in conjunction with other micturition control methods.

## 1. Introduction

Optogenetics, a technology that enables precise and specific modulation of cell activation or deactivation with high temporal and spatial resolution using light [1,2,3,4], has been widely utilized in various biomedical engineering research endeavors. For example, ongoing research directly targets neurons within the central nervous system for treating conditions such as depression [5,6,7] and Parkinson’s disease [8,9], or to rehabilitate damaged neurons through repeated stimulation [10]. Moreover, studies are in progress to regulate the function of various organs (such as the lungs [11,12], gastrointestinal tract [13,14], and penile for erection [15]) by manipulating cellular activity within these organs or by interacting with their connected neurons. One of the most active research fields that utilizes optogenetics is vision restoration, which was the first to receive clinical trial approval from the FDA. After receiving the official approval for the treatment of visual impairments, this field is rapidly expanding its research into clinical applications from the perspective of translational medicine [16]. In doing so, this field continues to advance existing research trends and explore the potential for new treatment methods. This represents a contrast to its early use in exploring neural networks in the field of neuroscience, where it served as an alternative or complementary option to other stimulation technologies. 

Recently, optogenetics has been applied in research for micturition control to modulate its complex neural networks. This includes targeting the central nervous system [17,18,19,20,21], the peripheral nervous system [22,23,24], and the bladder [25,26,27]. However, most of the results so far have focused on controlling bladder activity. There is a lack of results from optogenetics applications that specifically target the external urethral sphincter (EUS), which also plays a key role in micturition by cooperatively contracting and relaxing with the bladder during the storage and voiding phases, respectively [28].

During the phase of storing urine within the body, the contraction of the EUS not only prevents unexpected urine release, but also plays a crucial role in maintaining continence by triggering the activation of the secondary reflex nerve, a process that orchestrates bladder relaxation and the inhibition of voiding [29]. Hence, through the utilization of optogenetic techniques to stimulate the EUS muscle, we anticipate that it will contribute to stabilization of urinary incontinence through a process similar to the natural physiological mechanism in organisms. In particular, the optogenetic approach to the EUS is expected to carry even greater significance when the neural networks associated with micturition activities are abnormal, such as in cases of damage or severance. This technique holds potential to evolve into a more efficient and improved technology through integration with existing micturition control research.

In this study, we propose that optogenetics can be applied to modulate the EUS of *C57BL/6* mice, and that sensitized micturition activity can be stabilized by optical stimulation of the EUS’s striated muscle layer. For this purpose, we delivered the AAV (Adeno-associated virus) vector into the EUS tissue of the mice to induce the expression of the light-gated ion channels (hChR2/H134R) in the striated muscle cells. Subsequently, the EUS tissue of the urethane-anesthetized mice was stimulated with light delivered through optical fibers, and the activation of the muscle tissue was confirmed through electromyography (EMG). Ultimately, we verified that the sensitized micturition activity induced by acetic acid could be normalized through the optical stimulation of the EUS expressing channel rhodopsin.

## 2. Material and Method

### 2.1. Overview of Experiment

**Figure 1:**
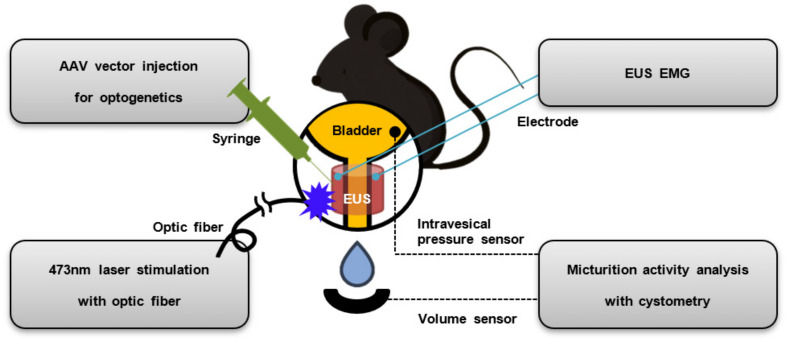
An overview of optical external urethral sphincter (EUS) activation in mice and the experimental procedure. The viral vector injection, insertion of electrodes for EMG measurement, and optical stimulation are directly performed on the EUS muscle tissue. The micturition activity of the mice in response to optical stimulation is monitored and analyzed by measuring the intravesical pressure of the bladder and the voiding volume.

Figure 1 provides an outline of the experimental methodology and procedures used in this study. All experimental procedures and setups involve surgical access to the bladder and EUS in anesthetized adult mice. The AAV vector is injected with a syringe into the EUS muscle of the wild-type mouse to express the light sensitive channel rhodopsin protein at the membrane of the EUS muscle cell for the application of optogenetics technology. The EUS expressing light-gated ion channels is optically stimulated using laser light transmitted through an optical fiber. The resulting electrical activity of the EUS muscle, induced by optical stimulation, is analyzed by EMG measurement conducted with electrodes inserted directly into the EUS muscle tissue. To evaluate the impact of artificial manipulation of the EUS muscle on micturition, real-time cystometry measurements are conducted using a pressure gauge connected to the inside of the bladder through a catheter. Additionally, a volume sensor measures the amount of urine voided by the experimental animals. Through these procedures, we assess the effects of optogenetic stimulation on micturition in our study.

### 2.2. Animals

In these experiments, we exclusively employed only wild-type adult male *C57BL/6* mice (Samtako, Osan, Republic of Korea) as the volume of the EUS was larger than that in females [30]. EUS EMG and Cystometry were conducted on mice over 8 weeks old (weighing 25~30 g), and the AAV vector was administered to the animals at 6 weeks of age. 

Throughout this study, to minimize the physical and mental discomfort experienced by our experimental animals, we managed them in a systematically managed breeding facility, allowing them free access to food and water following a 12 h day/night cycle. After surgical procedures, we continuously monitored the conditions of the animals according to relevant guidelines, administering analgesics and antibiotics as necessary. In particularly severe cases, we carried out humane euthanasia. All other procedures related to animal experimentation were also conducted under the approval of the institution’s ethical committee and in accordance with related guidelines.

### 2.3. AAV Vector Injection for Optogenetics

The AAV vector (*AAV2/8-CMV-hChR2(H134R)-EGFP*, KIST Virus facility, Seoul, Republic of Korea), with a titer of 1.21 × 10^13^ GC/mL, was used to express the light-gated ion channels (hChR2/H134R) in the EUS muscle of the wild-type mice. The mice were anesthetized with 1.5~2% Isoflurane (Ifran, Hana Pharm. Co., Ltd., Seoul, Republic of Korea) while resting on a heating pad (JD-OT-06DT, JEUNG DO BIO & PLANT Co., Ltd., Seoul, Republic of Korea) to maintain their body temperature. Before the procedure, their abdomens were meticulously shaved and disinfected with 70% alcohol, and a minimal laparotomy was performed to expose the EUS. A volume of 9 µL of the viral vector mixed with 1 μL of Fast Green (Sigma-Aldrich, St. Louis, MO, USA), for visualization to accurately target the tissue and avoid invading surrounding tissues, was injected at a rate of 0.5 µL/min into right and left side of the EUS, each half, using a 10 µL Syringe (#701, Hamilton, Reno, NV, USA), a 33-gauge needle (7803-05, Hamilton, Reno, NV, USA), and a syringe pump (KDS-310-PLUS, KD Scientific Inc, Holliston, MA, USA). After the injection, the incision site was meticulously sutured with 6-0 silk (AILEE Co., Ltd., Seoul, Republic of Korea) and disinfected with povidone-iodine (Firson Co., Ltd., Gwangju-si, Republic of Korea). During the recovery period, lasting over two weeks, the mice received appropriate care and attention, including administration of analgesics (Carprofen (Rimadyl^®^, Zoetis, Parsippany-Troy Hills, NJ, USA), 5 mg/kg, S.C.) and antibiotics (Enrofloxacin (Baytril^®^, Bayer, Leverkusen, Germany), 5 mg/kg, S.C.), as needed based on their condition.

### 2.4. Cystometry

The mice were anesthetized with urethane (1.3 g/kg, S.C.) and their anesthesia state was confirmed through a gentle toe pinch. Subsequently, a laparotomy was meticulously performed to expose the bladder, following the same procedure used for viral vector injection. Then, a polyethylene catheter (KN-392, Natsume Seisakusho Co. Ltd, Tokyo, Japan) connected to a pressure sensor (BLPR2, World Precision Instruments, LLC, Sarasota, FL, USA) and a syringe pump (PHD 2000, Harvard Apparatus, Holliston, MA, USA) was inserted into the dome region of the bladder. After 2 h of recovery time post-catheterization, PBS or PBS diluted with 0.1% Acetic acid was infused into the bladder at a rate of 3 mL/h. To measure the voiding volume of the mice, a force transducer (FORT10G, World Precision Instruments, LLC, Sarasota, FL, USA) with a plastic cup was employed. Real-time data collection of intravesical pressure of the bladder and the voiding volume was conducted at a sampling rate of 500 Hz using a data acquisition system (Digidata 1440a, Molecular Devices, San Jose, CA, USA).

### 2.5. EUS EMG

The electromyography of the EUS of ChR2 expressing mice and control mice was conducted after cystometry while maintaining the urethane anesthesia. The EUS was exposed using the same method employed for viral vector injection. For EMG recordings, two manually made, hook-shaped electrodes using stainless steel wire (790700, A-M Systems Inc., Sequim, WA, USA) were carefully inserted for the EMG into the left and right sides of the EUS, with a ground electrode placed in the abdomen for reference. The EMG signals were recorded using a biosensing board (OpenBCI) at a sampling rate of 250 Hz.

### 2.6. Optical Stimulation

For optical stimulation of the EUS in ChR2-expressing mice and control mice, a light pulse train with a duration of 10 ms and a frequency of 40 Hz was provided using a laser equipment (MBL-F/300mW, CNI Optoelectronics Tech, Changchun, China), optical fibers (0.37NA, Medlight SA, Ecublens, Switzerland), and a shutter system (SR470 and SR475, Stanford Research Systems, Sunnyvale, CA, USA). The intensity of the light was carefully monitored by a power meter (PM100D and S130C, Thorlabs, Newton, NJ, USA) and kept below 1.5 mW/mm^2^ by using a rotating ND filter (NDC-50C-4M-B, Thorlabs, Newton, NJ, USA) throughout all experiments. The end of the optical fiber was placed directly in front of the exposed EUS. The timing of stimulation was manually controlled; the starting point was determined based on parameters of repetitive micturition activity (such as the average micturition period and threshold pressure), and the ending point was determined by the onset of voiding.

### 2.7. Immunofluorescence

The extracted EUS from the mice was pre-fixed in 4% PFA for 12 h and cryoprotected in 30% sucrose for more than 24 h. The EUS tissue was rapidly frozen with OCT compound (FCS 22 Clear, Leica Biosystems, Germany) and was cryo-sectioned to obtain 40 µm-thick slices (CM1950, Leica Biosystems, Nußloch, Germany). Subsequently, the sectioned tissue samples were permeabilized for 30 min in 0.1% Triton X-100 and treated with 10% bovine serum albumin (BSA) for 1 h to minimize non-specific reactions. To detect an alpha-actinin, the primary antibody (Anti-alpha-actinin antibody, Abcam, Cambridge, UK) was applied to the samples at a ratio of 1:100 with 4% BSA and left overnight at 4 °C. For further visualization, the secondary antibody (Goat Anti-Rabbit IgG H&L (Alexa Fluor^®^ 594), Abcam, UK) was applied to the samples at a ratio of 1:100 with PBS for 3 h at 4 °C. Finally, the nuclei were stained with Hoechst dye (R37605, Invitrogen, Waltham, MA, USA) for 15 min, and the tissues was mounted with a fixing solution (HISTOMOUNT, Life Technologies, Carlsbad, CA, USA). The samples were observed at 10× and 40× magnifications using a confocal fluorescence microscope (TCS SPE, Leica Microsystems, Wetzlar, Germany).

### 2.8. Data Analysis

The values and event timings for peak intravesical pressure, baseline pressure, the voiding volume, and the micturition period from the cystometry were obtained using the software of the data acquisition system, pClamp 10.7.0. Subsequently, the collected data of micturition activity and EMG signals were analyzed and visualized using OriginPro 9. To assess the statistical significance of the micturition activity data, mixed-effects models were employed, and the analysis was conducted using R (v4.1.2) with the lme4 package (v1.1-18-1).

## 3. Result

### 3.1. Histological Examination of EUS for the Evaluation of Optogenetic Approaches 

**Figure 2:**
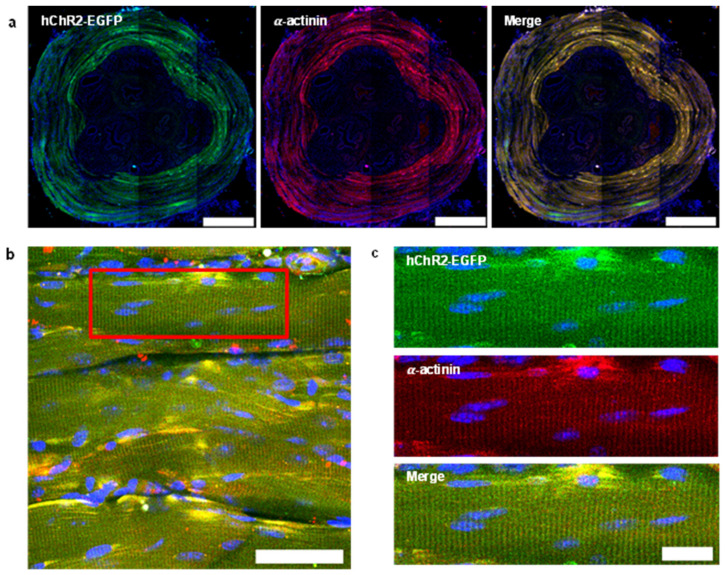
Results of Immunofluorescence and confocal microscopy following AAV vector injection into the EUS muscle of mice. The nucleus of the striated muscle cell (blue), hChR2-EGFP (green) and alpha-actinin (red) are selectively combined and displayed. The cross-section of the proximal EUS obtained through the integration of 10× captured images (**a**). An image of the striated muscle cells of the EUS (**b**). An enlarged view of the indicated red box (**c**). Scale bars: (**a**) 500 µm; (**b**) 50 µm; (**c**) 20 µm.

We employed an AAV vector composed of the AAV2/8 serotype and CMV promoter, both of which exhibit high expression efficiency in striated muscle cells [31], to introduce the light-gated ion channels and fluorescent protein (hChR2-EGFP) into the striated muscle cells of the EUS in wild-type mice. To evaluate the expression, we performed Immunofluorescence with an anti-alpha-actinin antibody, which specifically marks striated muscle cells, and examined the samples using a fluorescent confocal microscope. Upon integrating multiple images captured at 10× magnification from cross-sectioned tissue, we verified the presence of hChR2-EGFP expression, distributed along the circumference of the EUS (Figure 2a). Fluorescent signals were observed to be quite selectively distributed within the striated muscle cell region of the EUS while such signals were not found in other areas. This selective localization of the expression was possible despite the CMV promoter in the viral vector, known for inducing ubiquitous expression. This was made possible due to the precise and controlled insertion of the viral vector directly into the muscle tissue, effectively preventing any indiscriminate spread. Furthermore, upon examining the 40× magnification images and their enlargements, we could observe the distribution of hChR2-EGFP, which could only be confirmed in the subject where the viral vector was injected (see Figure S1), and alpha-actinin, revealing the intricate structure of the T-tubule system and Z-disk within the striated muscle. This analysis unequivocally confirmed that the expression of these proteins localized to the membrane (Figure 2b,c).

### 3.2. EUS EMG in Response to Optical Stimulation

**Figure 3:**
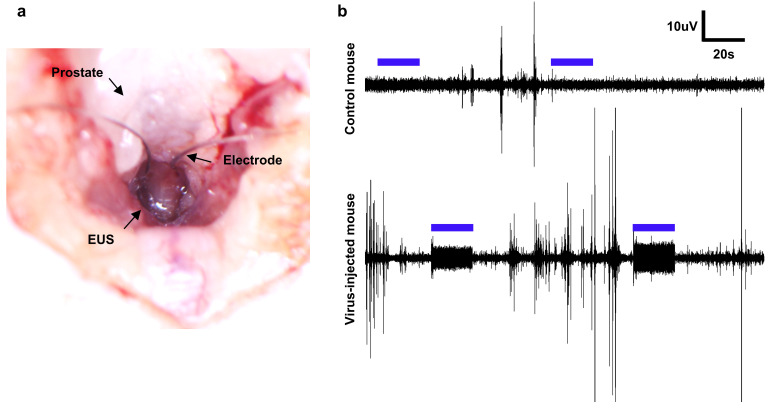
Results of electrode insertion for EUS EMG measurements (**a**). EUS EMG responses to optical stimulation (blue line) recorded in the control and experimental mice (**b**).

To verify the optical activation of the EUS, we conducted EMG on urethane-anesthetized mice during the resting period of micturition activity (without PBS infusion) to avoid the overlap of signals from spontaneous muscle activity with those resulting from the activity induced by optical stimulation. For this purpose, hook-shaped electrodes and optical fiber tips were strategically positioned at the same locations where the AAV vector was injected (Figure 3a). The muscle tissue was then stimulated with a laser at a wavelength of 473 nm, a duration of 10 ms and a frequency of 40 Hz pulse train. The protocol for optical stimulation applied in this study was adopted based on the findings presented in the preceding research [32]. The EMG results show that only the mice in the experimental group exhibited electrical responses to optical stimulation, and the activity of the EUS was sustained for at least 20 s. In contrast, in the control mice, electrical activity was not detected in response to the pulsed optical stimulation given continuously for 20 s. This demonstrates that the thermal effects from optical stimulation, which could potentially generate unexpected side effects in the tissue, are absent. Additionally, it confirms that the photoelectric effects that might arise from the electrodes do not induce unnecessary noise during EMG measurements. Spontaneous EMG signals were also observed in both the control and experimental mouse. The magnitude of the electrical signals induced by optical stimulation (~20 µV_pp_) was relatively smaller compared to the signals from spontaneous contractions (~100 µVpp). However, considering that the stimulation and EMG measurements of the EUS were locally performed on the proximal side within the elongated cylindrical EUS tissue, this is still regarded as a normal level (Figure 3b).

### 3.3. Cystometry Analysis Demonstrating the Relaxation of Sensitized Micturition

**Figure 4:**
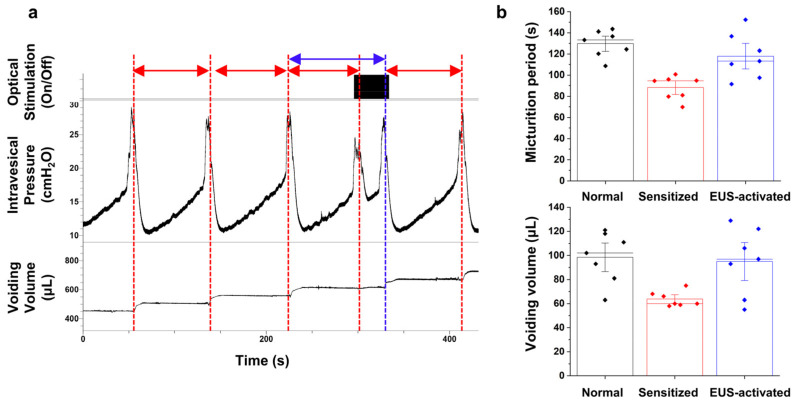
The representative results of cystometry (**a**), along with the statistical analysis (**b**) in which seven micturitions are considered for each condition from a single experimental mouse. It was observed that the micturition activity, sensitized by acetic acid (red arrows and bars), was stabilized to a level similar to normal micturition activity due to EUS activation (blue arrows and bars). All error bars indicate the SE.

Finally, cystometry was conducted on urethane-anesthetized mice to demonstrate the alleviation of sensitized micturition activity by optical EUS activation. In order to induce normal and sensitized micturition activity, PBS and acetic acid diluted to 0.1% in PBS were sequentially infused into the bladder through the catheter. Optical stimulation was applied during the acetic acid-induced sensitized micturition activity, synchronized with the expected timing of the following micturition, as determined through statistical analysis. Figure 4a illustrates the typical response of micturition activity during consecutive voidings in relation to optical stimulation. The section indicated by the red arrow shows a shortened micturition period due to the influence of acetic acid. On the other hand, the section pointed out by the blue arrow illustrates a temporary delay (stabilization) of the micturition due to optical stimulation. If optical stimulation is no longer applied after the delayed micturition, it has been confirmed that the micturition period returns to the state before stimulation without any significant side effects. Figure 4b presents cystometry analyses of micturitions from a single subject. The micturitions are categorized as ‘Normal’ (micturition with PBS infusion, without stimulation), ‘Sensitized’ (micturition with 0.1% acetic acid infusion, without stimulation), and ‘EUS-Activated’ (micturition with 0.1% acetic acid infusion, with stimulation), based on the conditions applied to the subject. The normal micturition period (129.7 ± 12.6 s) and the voiding volume (98.4 ± 21.0 µL) were shortened to a reduced period (88.1 ± 11.3 s) and decreased volume (63.7 ± 6.2 µL) due to acetic acid. Following this, optical EUS activation was applied, resulting in the stabilization of the sensitized micturition activity, leading to an extended micturition period (117.8 ± 21.4 s) and increased the voiding volume (95.0 ± 27.8 µL). 

**Figure 5:**
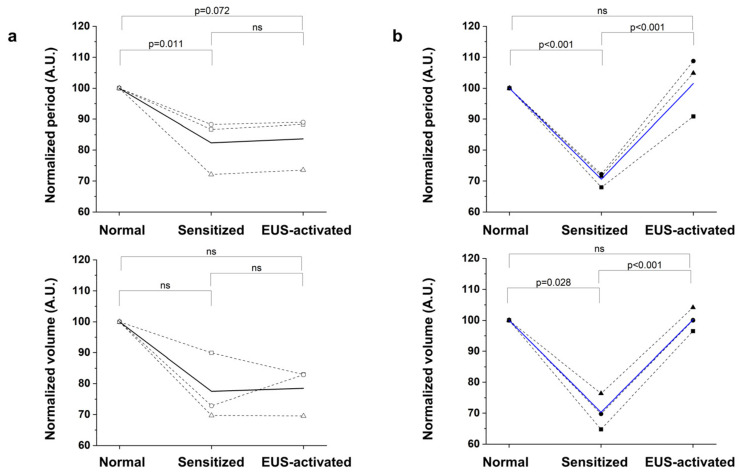
Results of optical stimulation and micturition activity analysis of the control group ((**a**), wild-type animals, *n* = 3) and the experimental group ((**b**), AAV-injected animals, *n* = 3). The changes for each individual (dash line) and their average (solid line) are shown. ns: stands for ‘not significant’.

Based on the results obtained from a single subject, we conducted repeated experiments for both the control and experimental groups and carried out statistical analyses on these. In an effort to minimize the number of animal subjects, we used three animals per group in this process. The statistics results show consistency across all experimental subjects, unlike the control subjects where no changes in micturition activity were observed in response to optical stimulation. Figure 5a presents the results from the control group consisting of wild-type mice without the viral vector inserted, while Figure 5b shows the results from the experimental group with the viral vector inserted. The results were normalized to determine relative values against normal micturition. The average values of the micturition period and the voiding volume of the experimental subjects, which initially decreased to 70.58% and 70.27%, respectively, due to sensitized micturition activity, were restored to 101.49% and 100.22%, respectively, after activation of the EUS. To maximize statistical power by utilizing every individual datapoint, mixed-effects models were adopted to assess statistical significance of our data. The model confirmed that the optical stimulation significantly extended the micturition period (df = 45.1, t = 6.684, *p* < 0.001) and increased the voiding volume (df = 45.2, t = 4.371, *p* < 0.001), which suggests it stabilized the sensitized micturition activity. Due to the small sample size, there were parts in the control group data graph where statistically significant levels of *p*-values were not achieved; however, it was confirmed that in the experimental group data where optogenetics was applied, it nonetheless demonstrated a sufficient level of statistical significance.

## 4. Discussion

Experimental results demonstrated that, after the insertion of an AAV vector into the EUS of wild-type mice and a recovery period of at least two weeks, there was sufficient expression of light-responsive proteins in the EUS, and these proteins could induce electrical activity in the muscles upon optical stimulation, as assessed via histology and EMG measurements. Furthermore, it was demonstrated through cystometry measurements and analyses that this activation of the EUS muscles by optical stimulation could restore the sensitized micturition reactions caused by acetic acid to a state close to normal.

As seen in the EMG results, the amount of EUS activity generated by optical stimulation to relax the sensitized micturition response was smaller compared to spontaneous EUS activities (see Figure S2). This finding suggests that continuously low-level activation of the EUS through optical stimulation can be a highly effective approach for micturition control, especially considering the EUS’s tendency to fatigue quickly [33].

In the cystometry analysis, aside from the micturition period and the voiding volume, no significant differences were observed in various parameters between the experimental and control groups. Particularly, symptoms that could occur due to non-coordinated contraction of the EUS such as increased peak intravesical pressure and baseline pressure, were not observed (Table 1) [34]. This indicates that the effect of bladder relaxation resulting from the activation of secondary reflex nerves, rather than the effect of interrupting urine flow due to EUS contraction, contributed more significantly to the stabilization of micturition activity.

In this study, we employed the widely used hChR2(H134R), which required optical stimulation to be sustained for tens of seconds to achieve effective relaxation of micturition activity. This points to the need to enhance energy efficiency by reducing the energy input required for the stimulation and to optimize muscle activity in response to it. The immunofluorescence results currently demonstrate that hChR2 is sufficiently expressed in the striated muscle cells of the EUS. However, we anticipate that by refining the serotype and promoter of the viral vector, we could improve the transfection efficiency and reduce the required optical energy for the optogenetic micturition control. Improvements in the optical EUS activation could be achieved by using light-sensitive proteins that are better suited for controlling striated muscle cells. The adoption of switch-type proteins could potentially replace the need for continuous optical stimulation, thereby reducing energy consumption from stimulation devices [35]. Furthermore, it is anticipated that by applying an anion channel protein such as GtACR1 instead of a cation channel protein to the EUS, it could develop into a technology capable of addressing various micturition dysfunctions by inhibiting the contraction of the EUS muscle.

In small animals, it has been possible to control micturition by applying optogenetics to EUS tissue. However, applying this technology in medium to large animals can entail several difficulties. More substantial amounts of viral vectors may be needed to express opsin in larger volumes of muscle tissue, and there could be challenges ensuring that light stimulation penetrates deeply enough into the muscle layers to induce uniform and sufficient force to foster functional activity. These difficulties are still considered common limitations when applying optogenetics to muscle control, but it is anticipated that continuous advancements in viral vector manufacturing technology and the development of more improved red-shifted opsins will lead to better improvements.

Despite the fact that the viral vector injection and optical stimulation were limited to the proximal part of the EUS, we hold the belief that advancements in viral vector delivery systems and stimulation devices specifically tailored for the EUS could result in more effective control, including the distal part of the sphincter. Considering the challenges in accessing the distal EUS due to its curved path following the complex pelvic structure, there is a need for the development of devices better suited for this region. 

Moreover, if we perform such optimization in conjunction with a closed-loop system integrated with sensors to measure the status of the bladder (such as intravesical pressure and volume) or the urine flow through the urethra, it could offer even more refined control. These sophisticated systems have the potential to become valuable therapeutic tools for treating micturition disorders and could find applications in lower urinary tract research areas. Additionally, they could be utilized to create disease models such as Detrusor Sphincter Dyssynergia in experimental animals, and are thus expected to contribute in various ways.

## 5. Conclusions

In this study, we utilized optogenetics to activate the external urethral sphincter in mice, and our findings demonstrate that optical stimulation of the external urethral sphincter leads to relaxation of sensitized micturition activity, resulting in extended micturition periods, and increasing voiding volumes.

These results hold great promise as they can be applied either independently or in conjunction with various other technologies aimed at regulating micturition activity in the bladder and external urethral sphincter. Furthermore, they are expected to significantly contribute to the advancement of biomedical engineering technologies based on optogenetics. 

## Figures and Tables

**Table 1:** Analysis of cystometry with parameters of peak intravesical pressure and baseline pressure, comparing the experimental and control groups with and without optical stimulation.

		w/o Stimulation		w/Stimulation	
		Intravesical Pressure (cmH_2_O)	Baseline Pressure (cmH_2_O)	Intravesical Pressure (cmH_2_O)	Baseline Pressure (cmH_2_O)
Control group	1	38.7 ± 1.5	10.6 ± 0.6	40.2 ± 1.6	5.4 ± 0.1
	2	40.3 ± 0.9	6.4 ± 0.2	37.9 ± 1.0	5.7 ± 0.4
	3	29.8 ± 1.3	7.7 ± 0.2	26.8 ± 0.6	8.4 ± 0.7
Experimental group	1	32.7 ± 2.2	8.3 ± 0.3	34.0 ± 2.7	8.0 ± 0.5
	2	26.4 ± 0.8	8.0 ± 0.4	27.0 ± 1.3	7.0 ± 0.8
	3	28.8 ± 1.2	7.0 ± 0.8	32.4 ± 2.3	5.4 ± 0.1

## Data Availability

The original data supporting the findings of this research are available from the corresponding author upon request.

## Supplementary Materials

The following supporting information can be downloaded at: https://www.mdpi.com/article/10.3390/life13101961/s1, Figure S1: Immunofluorescence and confocal microscopy images for comparing the control and the experimental animal; Figure S2: Results of EUS EMG with wild type mouse over extended durations.
