# Supplementary material for: Optical EUS Activation to Relax Sensitized Micturition Response
Source: Life (Basel). 2023 Sep 25;13(10):1961. doi: 10.3390/life13101961 (PMC10608351; doi:10.3390/life13101961)
Supplement: Supplementary file 1 [file life-13-01961-s001.zip › life-2573212-supplementary.pdf]

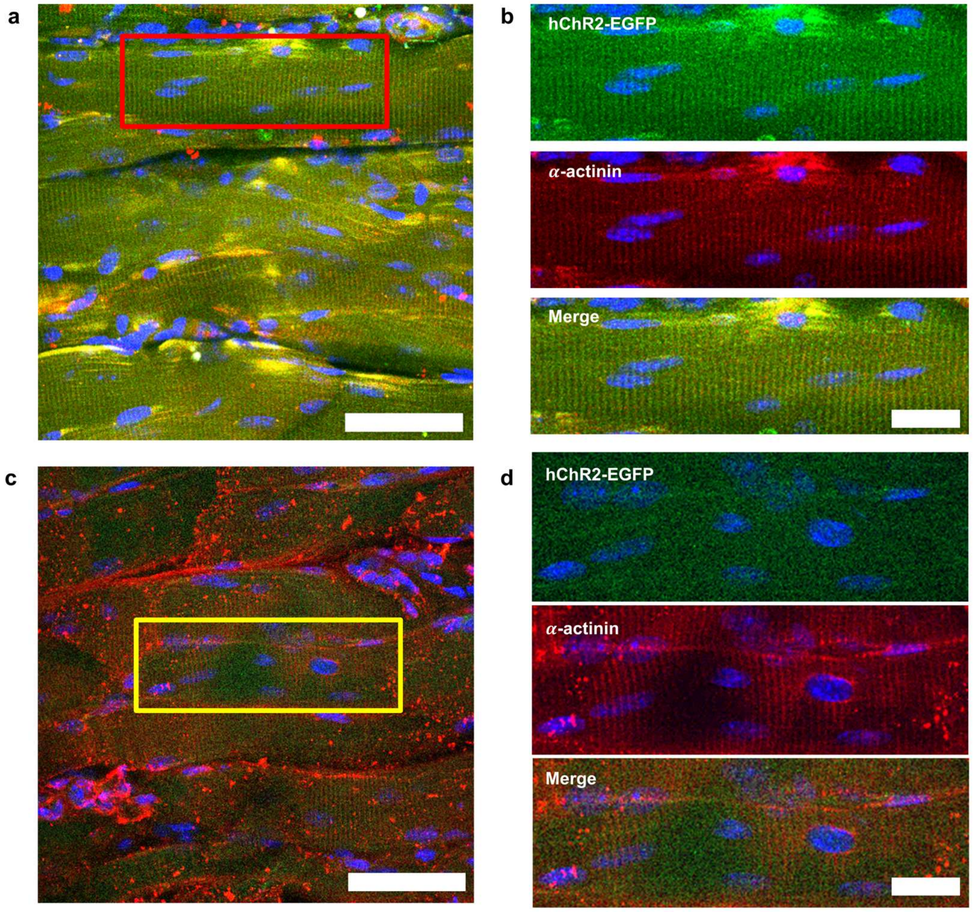

Figure S1. Immunofluorescence and confocal microscopy images for comparing the control and the experimental animal. Images of the striated muscle cells of the EUS (**a**, **c**) and enlarged views of the indicated red and yellow box (**b** and **d**, respectively). Only the result of experimental animal (**a**, **b**), where the viral vector has been inserted, shows the hChR2-EGFP fluorescence signal along the T-tubule system. The signal output of hChR2-EGFP from the control animal (**c**, **d**) appears to be predominantly background noise. Scale bars: (**a**) and (**c**), 500μm; (**b**) and (**d**), 50μm.

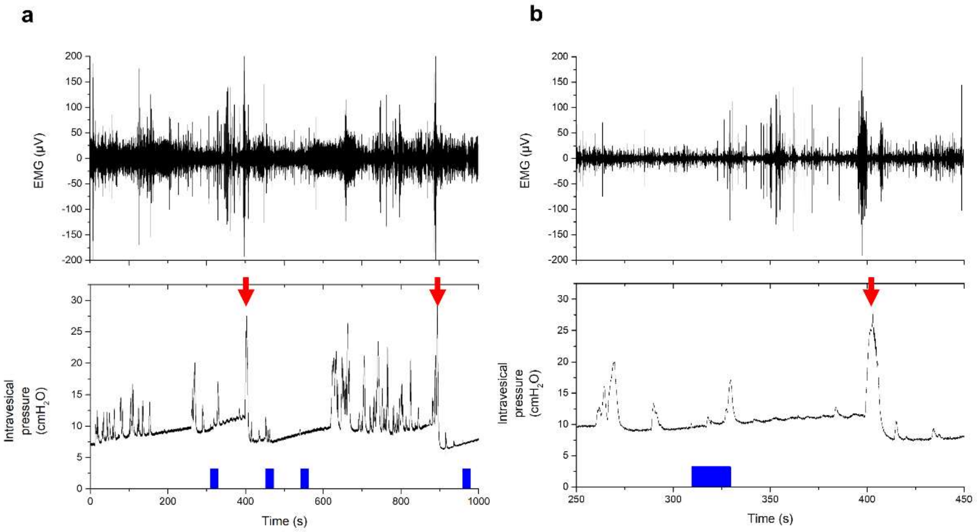

Figure S2. Results of EUS EMG with wild type mouse over extended durations. While infusing saline at a slow rate (1ml/hour) into the bladder of a wild type mouse where no virus was inserted, we measured the intravesical pressure and EUG EMG signals during 1000s (**a**) and 200s (**b**), and attempted a 20-second pulse train optical stimulation (blue bar) at a random moment. Please note that the amplitude of the EUS EMG occurring during micturition activity often exceeds 150 μV. Red arrow indicates the moment when micturition starts.
